# Lung nodule detected by F-18 fluorodeoxyglucose positron emission tomography-computed tomography in patients with papillary thyroid cancer, negative ^131^I whole body scan, and undetectable serum-stimulated thyroglobulin levels: two case reports

**DOI:** 10.1186/1752-1947-6-374

**Published:** 2012-10-31

**Authors:** Chan-Hee Jung, Hyeon-Jeong Goong, Bo-Yeon Kim, Jung-Mi Park, Jeong-Ja Kwak, Chul-Hee Kim, Hyun-Sook Hong, Sung-Koo Kang, Ji-Oh Mok

**Affiliations:** 1 Department of Internal Medicine, Division of Endocrinology and Metabolism, Soonchunhyang University School of Medicine, Bucheon, Korea; 2 Department of Nuclear medicine, Soonchunhyang University School of Medicine, Bucheon, Korea; 3 Department of Pathology, Soonchunhyang University School of Medicine, Bucheon, Korea; 4 Department of Radiology, Soonchunhyang University School of Medicine, Bucheon, Korea; 5 Division of Endocrinology and Metabolism, Soonchunhyang University School of Medicine, #108 Jung-Dong, Wonmi-Ku, Bucheon, Kyunggi-Do, 110-746, Korea

**Keywords:** F-18 fluorodeoxyglucose positron emission tomography-computed tomography, Papillary thyroid carcinoma, Pulmonary nodule, ^131^I whole body scan

## Abstract

**Introduction:**

When a pulmonary nodular lesion is detected by F-18 fluorodeoxyglucose positron emission tomography-computed tomography in a patient with post-surgical papillary thyroid carcinoma with undetectable serum-stimulated thyroglobulin levels and negative ^131^I whole body scan, diagnosis and management of the nodule may be confusing.

**Case presentation:**

We describe two post-surgical patients with papillary thyroid carcinoma who showed pulmonary nodular lesions detected by F-18 fluorodeoxyglucose positron emission tomography-computed tomography. In both cases serum-stimulated thyroglobulin levels were undetectable and nodular lesions were not detected by ^131^I whole body scan. In the first case, a 64-year-old Asian woman showed one focal increased fluorodeoxyglucose uptake lesion in the right lower lobe of one of her lungs. Based on the histologic study, the pulmonary nodular lesion was diagnosed as a solitary pulmonary metastasis from papillary thyroid carcinoma. In the second case, a 59-year-old Asian woman showed a new pulmonary nodule in the right lower lobe. The computed tomography scan of her chest revealed a 9mm nodule in the anterior basal segment and another tiny nodule in the posterior basal segment of the right lower lobe. Six months later, both nodules had increased in size and miliary disseminated nodules were also seen in both lungs. Based on their histology, the pulmonary nodular lesions were considered to be primary lung adenocarcinoma.

**Conclusions:**

The present cases emphasize that physicians should be cautious and make efforts for an accurate diagnosis of pulmonary nodules detected on F-18 fluorodeoxyglucose positron emission tomography-computed tomography in patients with papillary thyroid carcinoma with no evidence of metastasis such as negative ^131^I whole body scan and undetectable stimulated serum thyroglobulin levels.

## Introduction

The detection of a pulmonary nodule in patients who have papillary thyroid carcinoma (PTC) often presents us with a diagnostic dilemma because the lung is one of the most common sites of metastasis of PTC. However, primary lung cancer [1] and benign disease entities, including nonspecific findings, can also coexist with PTC. Radioiodine scans and serum thyroglobulin (Tg) measurements are the most commonly used methods for monitoring patients with thyroid cancer [2]. F-18 fluorodeoxyglucose positron emission tomography (FDG-PET) is primarily used to detect recurrence or metastatic disease in patients who have negative findings with ^131^I whole body scan (WBS) and elevated Tg levels [3].

Although the clinical value of FDG-PET for the follow up of patients with thyroid cancer remains controversial, its use is rapidly increasing in clinical practice due to concerns about recurrence or metastasis of thyroid cancer. Also, some studies have indicated that FDG-PET is effective in patients with undetectable Tg levels [4,5].

The detection by FDG-PET of a pulmonary nodule in patients with PTC, even those displaying undetectable stimulated Tg and negative ^131^I WBS, includes a considerable variety of entities, therefore the diagnosis must be made with caution. Although solitary metastasis to the lung from thyroid cancer is quite rare, metastasis from PTC can manifest as a solitary pulmonary nodule. However, clinicians should be aware of the possibility that pulmonary nodules mimicking metastasis on FDG-PET with computed tomography (CT) in patients who have PTC could be primary lung cancer.

We describe two patients who presented with pulmonary nodular lesions by FDG-PET-CT and unexpected results on histologic confirmation with no evidence for distant metastasis on serum-stimulated Tg and ^131^I WBS.

## Case presentation

### Case one

**Figure 1:**
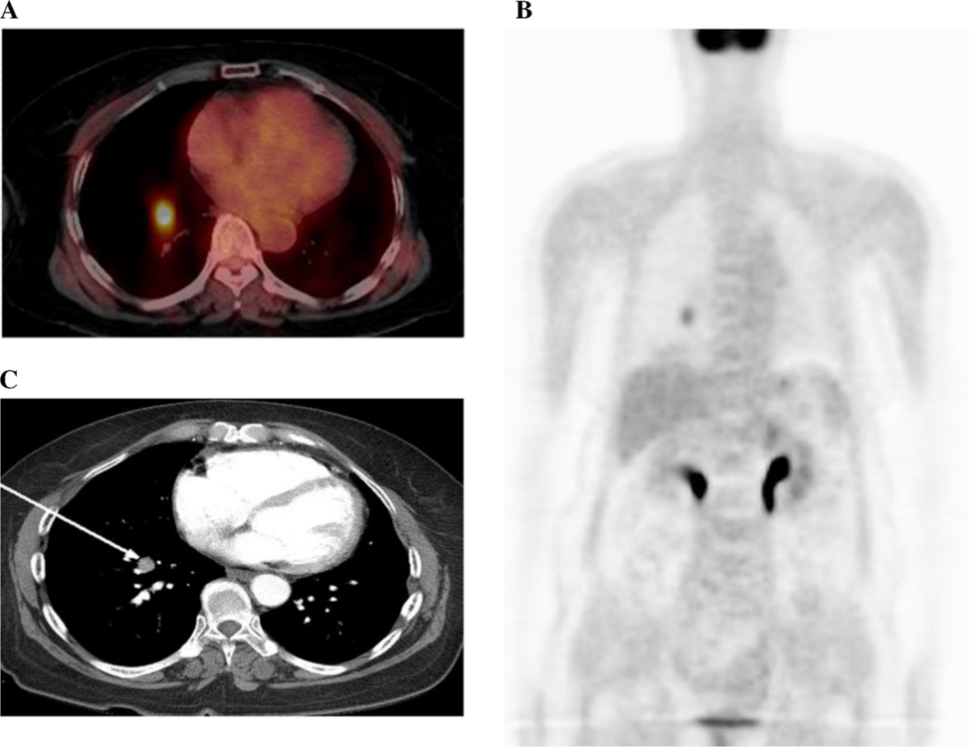
**The F-18 fluorodeoxyglucose (FDG) positron emission tomography-computed tomography (CT) scan (A: axial view, B: coronal view) shows a focal increased FDG uptake lesion (maximum standardized uptake value, SUVmax = 6.5) (A, B).** Chest CT scan shows an enhancing nodule measuring 1.3cm in the anterobasal segment of the right lower lobe (arrow) (**C**).

**Figure 2:**
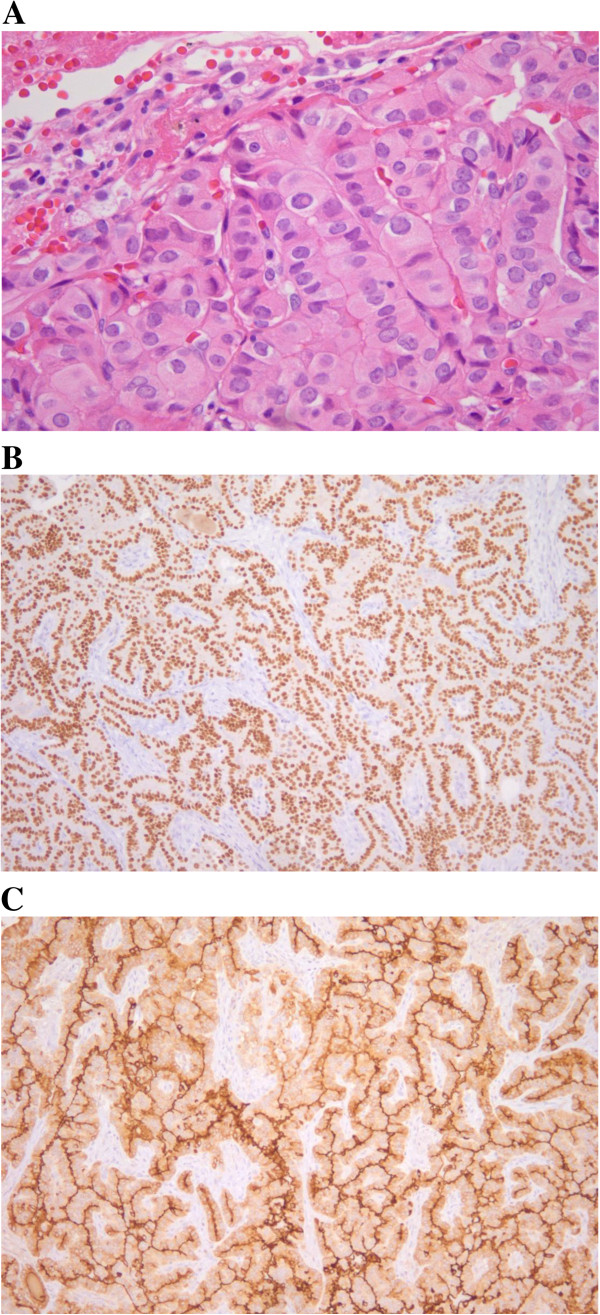
**The tumor cells have large round to oval pale nuclei, intranuclear inclusion, intranuclear groove and abundant cytoplasm (hematoxylin and eosin × 400) (A).** Immunohistochemical stain of thyroid transcription factor-1 reveals diffuse strong positivity in nuclei of tumor cells (**B**). Immunohistochemical stain of thyroglobulin shows positive result in cytoplasm of tumor cells (**C**).

A 64-year-old Asian woman underwent total thyroidectomy and 150mCi ^131^I therapy due to micro-PTC. The FDG-PET-CT images showed one focally increased FDG uptake lesion (maximum standardized uptake value, SUVmax = 6.5) in the right lower lobe (RLL) of one of her lungs 1 year after the operation and iodine ablation therapy (Figure 1A, 1B). The lesion was a 1.3cm nodule shown in a chest CT (Figure 1C). WBS showed no abnormal uptake and stimulated serum Tg (Tg level 0.79ng/mL, Tg antibody (Ab) 0.1U/mL) was undetectable. Because the tumor was located in the lower center of the horizontal fissure, it could not be approached via bronchoscopy or percutaneous transthoracic needle biopsy (PTNB). Thus, we performed a RLL lobectomy for definitive histologic diagnosis. A histologic examination revealed large round-to-oval pale nuclei, intranuclear inclusion, intranuclear groove and abundant cytoplasm (Figure 2A). Immunohistochemical staining of the cancerous cells was positive for cytokeratin (CK) 7, thyroid transcription factor-1 (TTF-1) and Tg (Figure 2B, 2C). Unexpectedly, these findings were consistent with a diagnosis of solitary pulmonary metastasis from PTC.

### Case two

**Figure 3:**
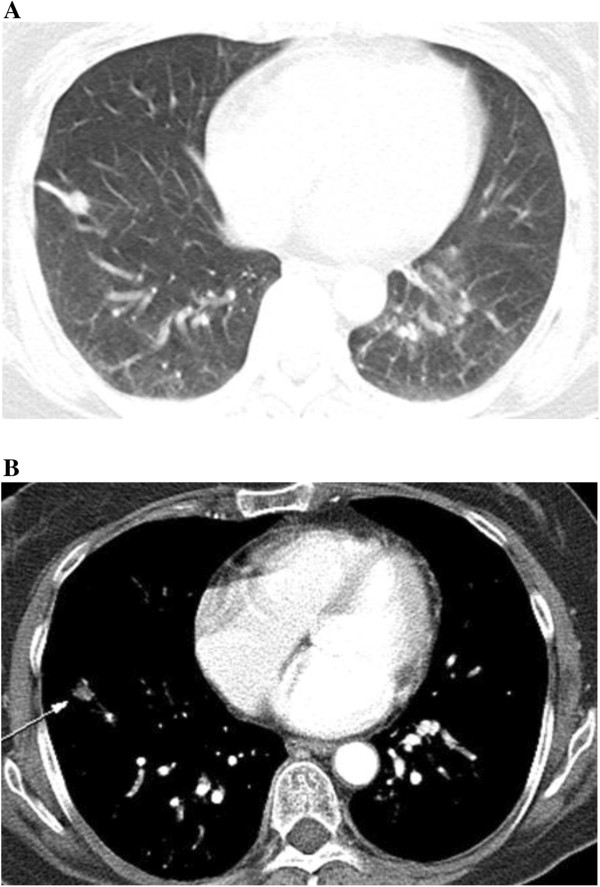
**The F-18 fluorodeoxyglucose positron emission tomography-computed tomography (CT) scan shows a newly appeared pulmonary nodule in the right lower lung (A).** CT scan shows a 9mm well-defined enhancing nodule in the anterobasal segment of the right lower lung (arrow) (**B**).

**Figure 4:**
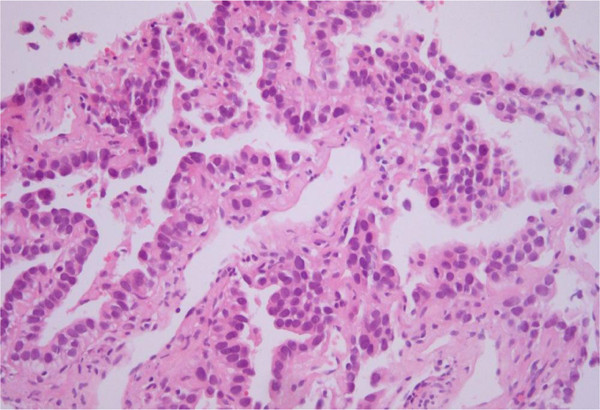
Needle biopsied lung tissue shows well-formed glandular structures lined by tumor cells with large hyperchromatic nuclei (hematoxylin and eosin × 200).

A 59-year-old Asian woman underwent a total thyroidectomy and 150mCi ^131^I ablation 4 years previously. There was no evidence for distant metastasis on a follow up ^131^I WBS and stimulated serum Tg level (Tg 0.32ng/mL, Tg Ab 0.02U/mL). However, a new pulmonary nodule was evident in the RLL on FDG-PET-CT (Figure 3A). Chest CT scans revealed a 9mm nodule in the anterior basal segment and another tiny nodule in the posterior basal segment of the RLL (Figure 3B). Six months later, these two nodules had increased in size and miliary disseminated nodules were present in both lungs. We performed CT-guided PTNB and transbronchial lung biopsy (TBLB) for differential diagnosis. Needle biopsied lung tissue showed well-formed glandular structures lined by tumor cells with large hyperchromatic nuclei (Figure 4). Based on the histology data, the pulmonary nodular lesions were considered primary lung adenocarcinoma. Immunohistochemical staining revealed the cancer cells as being positive for CK7 and TTF-1, but negative for CK20. The patient is receiving chemotherapy for primary lung cancer.

## Discussion

When a pulmonary nodular lesion is present in a patient with post-surgical thyroid cancer with undetectable stimulated Tg levels, diagnosis and management of the nodule can be confusing.

The typical radiographic appearance of pulmonary metastases from PTC is a random distribution of multiple nodules [6]. When a solitary nodule is present in patients with post-surgical PTC with negative ^131^I WBS and undetectable stimulated serum Tg levels, diagnosis of the nodule is likely to be primary lung cancer, not metastasis to the lung from PTC.

The first presented case represents a rare example of metastasis of PTC presenting as a solitary pulmonary nodule without a clinically suspicious metastasis. The patient initially posed a diagnostic problem because the lung mass was located in a site that was not easily accessible to biopsy. Therefore, we performed surgical resection for accurate diagnosis and treatment under an initial clinical impression of primary lung cancer.

Solitary metastasis to the lung from thyroid cancer is quite rare and 14 cases of a solitary pulmonary metastasis from thyroid cancer, including our case, have been reported [7,8]. Moreover, metastasis from micro-PTC has been reported in only five cases, including our case [7]. Seven of the 14 cases initially presented as a solitary lung nodule without a known history of thyroid cancer, with the remainder detected during follow-up for PTC.

In general, serum Tg measurement and radioiodine WBS are recommended for the postoperative follow-up of PTC [2]. However, metastatic diseases for which the ability to take up radioactive iodine is lost are possible. Nakada *et al*. reported that two of four patients with a solitary pulmonary nodule on multidetector-CT were proven to be metastasis from PTC but neither showed positive radioiodine uptake. Moreover, seven of nine patients (78%) having pulmonary metastasis showed negative radioiodine uptake [9].

CT using iodine-rich contrast material can affect the results of radioiodine WBS. However, we performed a chest CT scan after T4 withdraw of radioiodine WBS in both cases. Therefore, we think that the results of WBS were not affected by the contrast material of the chest CT scan.

For patients with non-iodine-avid disease, the detection of metastases may be delayed. Furthermore, it has been reported that stimulated serum Tg determinations most sensitively detect recurrence and metastasis. However, it should also be noted that even an undetectable stimulated Tg with negative Tg Ab, as in the present cases, does not exclude a metastatic focus.

Although more recent publications have provided data that support the use of FDG-PET scanning for indications beyond simple disease localization in Tg-positive, radioactive iodine scan-negative patients, the clinical application of FDG-PET in patients with thyroid cancer with stimulated Tg negative and radioactive iodine scan-negative are not recommended routinely. However, patients with cancer can receive medical costs benefits through the National Cancer Registration in Korea and costs of radiologic imaging studies are not expensive. Therefore, the use of FDG-PET-CT is rapidly increasing in real clinical practice in Korea. Wang *et al*. reported a sensitivity of 79.3% for PET in patients with a negative iodine scan, compared with only 18.6% sensitivity in those with positive iodine scan [10]. In the two presented cases, the pulmonary nodule in each patient was detected by chance. If we had not performed FDG-PET, detection of these nodules may have been delayed until other clinical clues developed.

The cause of a single metastasis in PTC is unknown. Although the mechanism is unclear, macronodular single metastatic foci and loss of capability in the uptake of radioiodine (functional loss of iodine uptake) is responsible for an evident worsening in prognosis.

However, as highlighted by the second case, pulmonary nodules in patients who have PTC can be primary lung cancer. Although the results of ^131^I WBS and serum Tg were negative, several nodules of lung were thought initially to represent a metastatic PTC due to a typical finding for metastasis of PTC. However, pulmonary nodular lesions were identified as pulmonary adenocarcinoma through PTNB and TBLB. In general, because primary lung cancer carries a poorer prognosis than PTC, if physicians consider multiple lung nodules as metastatic PTC, then inappropriate treatment may worsen prognosis.

The accurate diagnosis of a pulmonary nodule in patients with PTC is important in the choice of the optimal treatment. Radioactive iodine has been the mainstay of treatment for patients with distant metastases; young patients having iodine-avid pulmonary micrometastases achieved remission rates of 90% at 10 years [1]. However, for patients with metastatic disease for which the ability to take up radioactive iodine is lost, survival is poor (5-year and 10-year survival rate of 29% and 10%, respectively) [11]. A study by Casara *et al*. reported that the oldest patients (mean age 61.9 years) showed a tendency of nonfunctioning and macronodule metastasis. For patients with pulmonary metastases that are large or nonresponsive to radioactive iodine, surgical resection can be considered [11].

The challenging issue in differentiated thyroid cancer is that there are patients with metastatic disease who can lose their radioiodine uptake and have negative stimulated serum Tg. A pulmonary nodule detected by FDG-PET in patients with PTC even showing negative for both stimulated serum Tg and ^131^I WBS includes a considerable variety of entities. Thus, a diagnosis must be made with caution.

## Conclusions

The present cases were considered to offer important information that physicians should be cautious and make efforts for an accurate diagnosis about pulmonary nodules on FDG-PET-CT in patients who have PTC with no evidence of metastasis such as negative ^131^I WBS and undetectable stimulated serum Tg levels.

## Abbreviations

Ab: Antibody; CK: Cytokeratin; FDG-PET-CT: F-18 fluorodeoxyglucose positron emission tomography-computed tomography; PTC: Papillary thyroid carcinoma; PTNB: Percutaneous transthoracic needle biopsy; RLL: Right lower lobe; TBLB: Transbronchial lung biopsy; Tg: Thyroglobulin; TTF-1: Thyroid transcription factor-1; WBS: Whole body scan.

## Competing interests

The authors declare that they have no competing interests.

## Authors' contributions

CHJ and JOM contributed to patients’ diagnosis and treatment. HJG and BYK participated in the literature review. JMP and HSH contributed to radiology-related issues and JJK contributed to histology-related issues. CHK and SKK participated in interpretation of the case. All authors read and approved the manuscript.

## Consent

Written informed consents were obtained from the two patients for publication of these case reports and any accompanying images. A copy of the written consents is available for review by the Editor-in-Chief of this journal.
